# Angio-Seal™ Embolization: A Rare Etiology of an Acute Distal Limb Ischemia

**DOI:** 10.1155/2017/9613863

**Published:** 2017-10-17

**Authors:** Glenmore Lasam, Joshua Brad Oaks, Jeffrey Brensilver

**Affiliations:** Department of Medicine, Overlook Medical Center, Summit, NJ 07901, USA

## Abstract

We herein report a serious vascular complication of diagnostic cardiac catheterization due to an embolization of an Angio-Seal closure device causing acute lower limb ischemia. The Angio-Seal was deployed via the right femoral artery following the catheterization which embolized several hours later to the right popliteal artery. Fogarty embolectomy restored perfusion to the right lower limb; however, compartment syndrome subsequently developed which required evacuation of a hematoma and repair of right popliteal artery.

## 1. Introduction

The Angio-Seal, an arterial closure device placed after percutaneous endovascular access, facilitates rapid hemostasis, quick recovery, and prompt ambulation. This clinical vignette describes an unusual complication after arterial closure device placement with resultant embolization and subsequent compartment syndrome.

## 2. Case Presentation

A 56-year-old woman with no prior peripheral vascular symptoms presented with exertional dyspnea and chest discomfort. She had a history of hypertension, hyperlipidemia, and hypothyroidism. Following an abnormal stress test, an elective cardiac catheterization demonstrated minimal coronary artery disease (30% stenosis of proximal right coronary artery and 20% stenosis of left anterior descending coronary artery). The right femoral artery site was closed with a 6-French Angio-Seal device. After monitoring for an hour, she was started on metoprolol for her coronary artery disease. She was deemed ready for discharge; but, upon standing, she became weak, dizzy, and nauseated. She was found to have a blood pressure of 90/40 mmHg and a pulse of 50. Her blood pressure improved to 130/70 mmHg upon laying her flat in bed.

**Figure 1:**
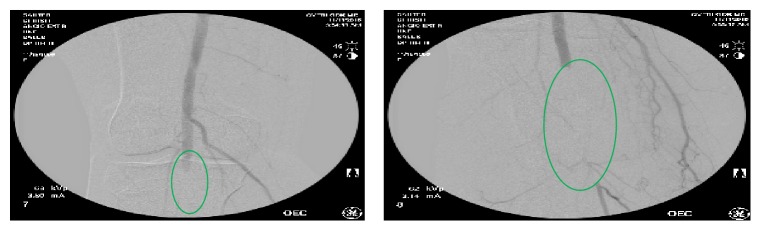
Intraoperative right leg angiogram showing below the knee filling defect at the right popliteal artery extending beyond the tibioperoneal trunk which likely represents a thrombus.

Orthostatic hypotension with symptomatic bradycardia was considered likely due to the beta blocker that was started after cardiac catheterization which was eventually discontinued. She was given a bolus of intravenous fluids and her discharge was held. Several hours later, she noticed that her right leg was cold and numb. Her blood pressure and heart rate have been within range during this time. The right dorsalis pedis and posterior tibial pulses were absent; she underwent an arteriogram, which revealed an abrupt cutoff of the right popliteal artery below the knee (Figure 1). Operative exploration of the popliteal artery was undertaken. A Fogarty embolectomy removed the thrombus and the maldeployed Angio-Seal closure device (in its native form) from within the arterial lumen restoring normal flow (with good back bleeding) to the arterial system. Palpable dorsalis pedis and posterior tibial pulses were noted thereafter. She was placed on full dose heparin and the wounds were closed. Several hours later, her pulses were again absent and she was emergently returned to surgery where she was found to have arterial compression from a hematoma in the popliteal fossa due to an anastomotic embarrassment of the popliteal artery suture line. The popliteal artery was repaired primarily, the hematoma was evacuated, and she was maintained on anticoagulation. She tolerated the procedure and was discharged on postoperative day number two.

## 3. Discussion

The conventional technique for maintaining hemostasis after femoral puncture is manual pressure on the incision site for at least ten to twenty minutes which can lead to extended immobilization and discomfort. Access site complications (1–5%) include bleeding, pseudoaneurysm formation, and vessel occlusion. This prompted device innovators to develop an alternative method to achieve hemostasis [1] that includes implantable collagen plugs and percutaneous suture devices [2].

The Angio-Seal device is a bioabsorbable arterial closure appliance that is deployed following a percutaneous intervention to attain hemostasis thus allowing early mobilization and reduced length of observation [3]. It is composed of a polylactide and polyglycolide polymer anchor, a collagen plug, and a suture [4]. The collagen plug should be pressed over the suture to achieve compression to the arterial external surface which in effect secures the anchor in the vessel lumen to entirely seal the puncture site. Ninety-five to a hundred percent of cases utilizing the Angio-Seal successfully achieved hemostasis [5]. Uncommon complications (0.5–1.9%) include infection, hemorrhage, and vessel occlusion [6, 7]. The dislodgement of the Angio-Seal in our vignette was likely linked to inadequate pressure applied to secure the collagen plug to the outer arterial wall during device deployment, allowing the intraluminal anchor to dislocate and dislodge to the popliteal artery, resulting in acute limb ischemia. Our clinical case is rare with only two reported cases of popliteal artery embolism caused by Angio-Seal closure device [8, 9] as well as a solitary case of distal embolic occlusion of the tibioperoneal trunk after an arterial closure device placement [10]. In our vignette, the patient`s hypotension with bradycardia was likely related to the beta blocker that was started after the procedure; however, the possibility of neurally mediated mechanism in the setting of presyncope triggered by the acute limb ischemia cannot be totally ruled out.

Arteriotomy was performed preferentially on the popliteal artery based on the arteriogram results that showed blockage at the site and not on the femoral artery. Fluoroscopically guided Fogarty embolectomy was utilized in our patient as in the other two reported cases of popliteal artery embolism because this allows for a directed embolectomy, thus minimizing intimal damage and distal embolization [8, 10].

Vascular closure device access site complications can be prevented through appropriate utilization of its indication as well as proper technique of insertion. Imaging modalities should be employed to confirm suspected vessel complications attributed to closure device. Frequent assessment of the puncture site and peripheral pulses is imperative to assess for evolving complications associated with percutaneous closure devices. It is possible that increasing the time of immobilization might reduce the likelihood of dislodgement of the closure device. Our clinical vignette emphasizes the importance of assessing the lower extremity arterial circulation both before and after an arterial based intervention. Maintaining a high index of suspicion and definitely intervening promptly and appropriately in such situations is critical to avoiding loss of limb functionality or limb itself.

## 4. Conclusion

A timely and accurate diagnosis is essential in all limb complaints particularly after placement of an arterial closure device. When recognized early, device-attributed complication can be managed successfully through simple and safe interventional technique.
